# Impact of prior cancer history on survival in brain malignancy: A propensity score‐adjusted, population‐based study

**DOI:** 10.1002/cnr2.1984

**Published:** 2024-02-22

**Authors:** Mohammad Ebad Ur Rehman, Fatima Faraz, Huzaifa Ahmad Cheema, Omer S. Ashruf, Hamna Raheel, Syeda Zainab Ali Naqvi, Nimrah Jabeen, Areesha Abid, Haris Mumtaz Malik, Ahmad Iftikhar, Ahmed Ibrahim, Sarya Swed

**Affiliations:** ^1^ Department of Medicine Rawalpindi Medical University Rawalpindi Pakistan; ^2^ Department of Medicine King Edward Medical University Lahore Pakistan; ^3^ Department of Internal Medicine Northeast Ohio Medical University Rootstown Ohio USA; ^4^ Department of Medicine Dow University of Health Sciences Karachi Pakistan; ^5^ Department of Medicine The University of Arizona Tucson Arizona USA; ^6^ Rawalpindi Medical University Rawalpindi Pakistan; ^7^ Faculty of Medicine University of Aleppo Aleppo Syria

**Keywords:** brain cancer, cancer‐specific survival, overall survival, prior cancer

## Abstract

**Background:**

Individuals with a Prior Cancer History (PCH) are often excluded from clinical trials. However, a growing body of evidence suggests that prior cancer history does not present adverse outcomes on cancer patients. The evidence on the survival of brain cancer patients in this regard remains widely unknown.

**Methods:**

We conducted a retrospective cohort study to estimate the prevalence and impact of prior cancer on survival of patients diagnosed with brain cancer. Data of patients who were diagnosed with brain cancer as their first or second primary malignancy between 2000 and 2019 were extracted from the Surveillance, Epidemiology, and End Results (SEER) database. Propensity Score Matching (PSM) was used to ensure comparable baseline characteristics among the patients. Survival analysis was conducted using the Kaplan–Meier method, as well as multivariate Cox proportional hazard and multivariate competing risk models.

**Results:**

Out of 42 726 patients, 1189 (2.78%) had PCH. Genitourinary (40.4%), Breast (13.6%), Hematologic and Lymphatic (11.4%), and Gastrointestinal malignancies (11.3%) were the most common types of prior cancer. PCH served as a significant risk factor for Overall Survival (OS) (Adjusted Hazard Ratio [AHR] 1.26; 95% CI [1.15–1.39]; *p* < .001) but did not have a statistically significant impact on Brain Cancer‐Specific Survival (BCSS) (AHR 0.97; 95% CI [0.88–1.07]; *p* = .54). Glioblastoma exhibited the most substantial and statistically significant impact on survival as compared to other histological types. Of all the organs systems, only prior Gastrointestinal and Hematologic and Lymphatic malignancies had a statistically significant impact on OS of patients.

**Conclusion:**

Our findings indicate that PCH does not exert a substantial impact on the survival of brain cancer patients, except in cases involving gastrointestinal or hematologic and lymphatic PCH, or when the brain cancer is glioblastoma.

## 1 INTRODUCTION

Primary brain and central nervous system (CNS) cancer is the 10th leading cause of cancer worldwide and the second leading cause of death in adolescents.^1^, ^2^ This malignant disorder is rare, accounting for only 1.3% of new cancer and 3% of deaths in all new cancer in 2022.^3^ However, the incidence of brain and CNS cancer is on the rise with a 94% increase in global cases and 76% increase in global mortality from 1990 to 2019.^4^ Poor survival in brain tumor patients may be attributed to stringent eligibility criteria that lead to poor clinical trial accrual.^5^ For instance, it is widely presumed that previous cancer treatment might interfere with the current outcomes and survival among patients. According to a Childhood Cancer study, the incidence of second primary cancer increased from 9% in 1975–1979 to 19% in 2005–2009.^6^ A dose‐related relationship between radio or chemotherapy during primary cancer has been associated with increased risk of certain second cancer types including CNS cancer.^6^ Second primary malignancy is associated with greater mortality and increased risk of other second primary cancers with some studies suggesting more than half the deaths occur in patients with second primary malignancy.^7^ The risk of second cancer varies considerably by type of first and second cancer, patient age, prevalence of second cancer, risk factors, primary cancer treatments, environmental and lifestyle exposures, and genetic susceptibility.^6^ A study by Murphy et al.^8^ evaluated the prevalence of prior cancer among newly diagnosed cancer patients in a population‐based study using the Surveillance, Epidemiology, and End Results (SEER) registry. They reported that approximately one‐fourth (25.2%) of older (≥65 years) adults and 11% of younger adults who were newly diagnosed with cancer had a history of prior cancer.

Currently, there is limited data analyzing the risk factors of developing primary brain cancer in patients with a previous history of cancer or evaluating the impact of prior cancer in patients with primary brain cancer. This study aims to highlight the impact of any previous history of cancer on the overall survival (OS) outcomes of primary brain cancer patients that are part of the SEER database. To date, there is no concrete data that proves that patients with a previous history of cancer have any impact on the outcomes in patients with a second primary cancer. Through this study, we hope to redefine clinical trial guidelines and increase the participation of patients with previous cancer that form a significant portion of cancer survivors. Increasing participation of this subgroup of brain tumor patients in clinical trials can help make the results of future clinical trials more generalizable.

## 2 METHODS

### 2.1 Database and case selection

**FIGURE 1:**
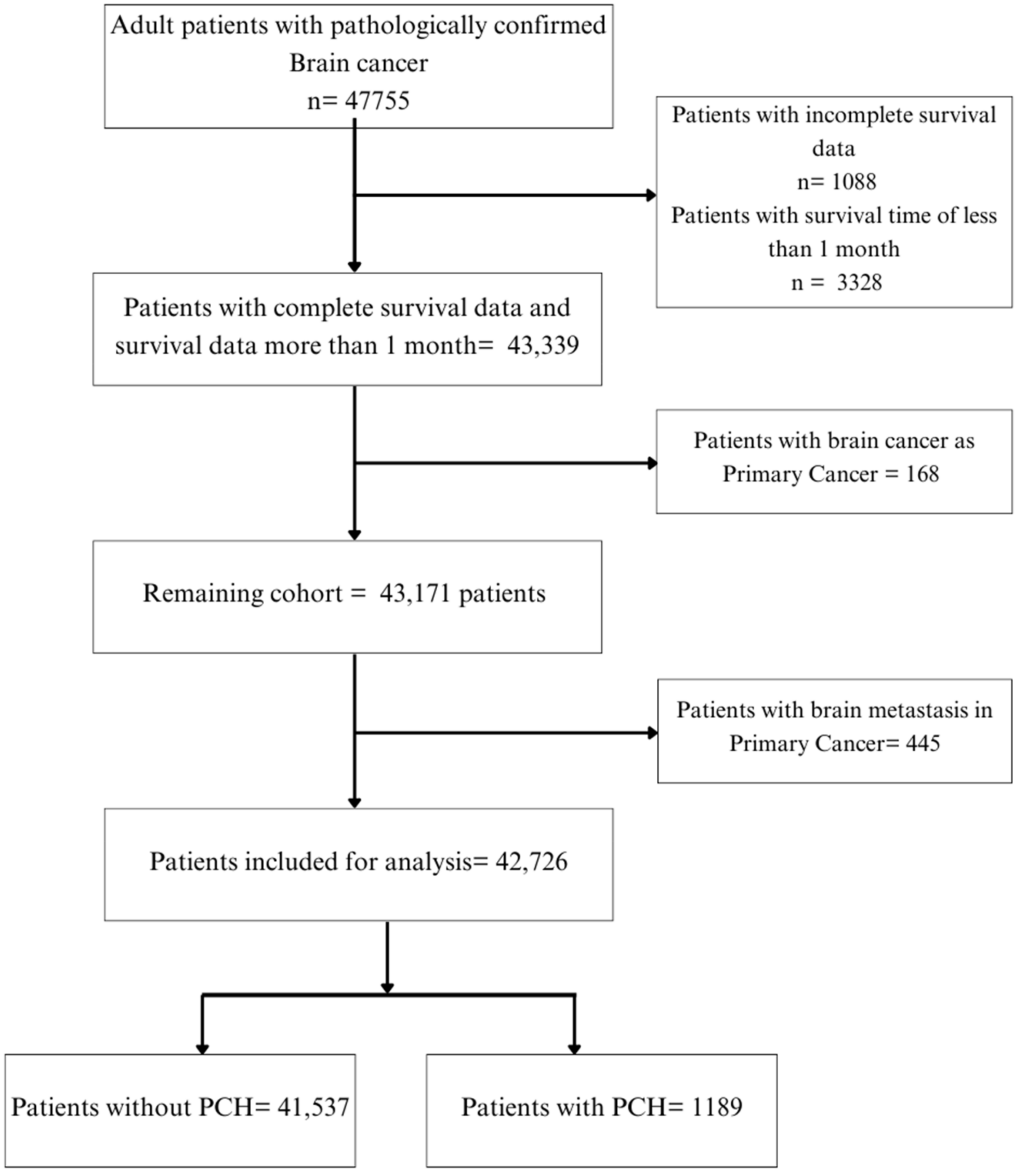
Flowchart demonstrating case selection process from SEER.

The SEER database, maintained by the National Cancer Institute, is the world's largest publicly available cancer database. All the data utilized in our study was extracted from the SEER database (Incidence—SEER Research Data, 17 Regs, Nov 2021 submission [2000–2019]) which collects cancer data covering approximately 26.5% of the US population, using the SEER*Stat software version 8.4.0.1. Patients diagnosed with primary brain malignancy (site codes C70.0‐C71.9) between 2010 and 2019 were included in this retrospective cohort study. Patients were included if they had no prior cancer history or only one prior cancer. A 2‐month time interval was necessary between the diagnosis of the first cancer and the diagnosis of brain malignancy to exclude synchronous primary malignancies. Patients who met the following criteria were excluded: (1) diagnosed on autopsy or death certificate records; (2) unknown follow‐up or survival data; (3) survival time less than 1 month; and (4) prior history of brain cancer. Finally, a total of 42 726 patients were included in our study; a detailed flowchart of case selection is present in Figure 1.

### 2.2 Study variables

**FIGURE 2:**
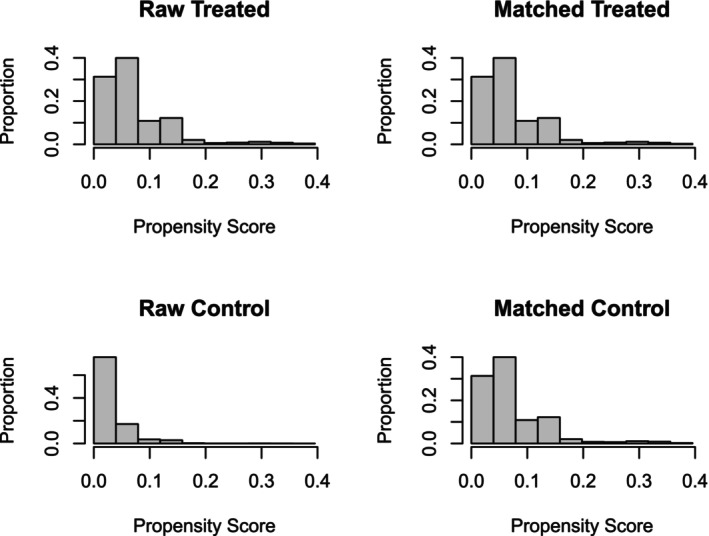
Propensity score matching.

We extracted demographic, pathological, and clinical data, including age at diagnosis, sex, year of diagnosis, race, marital status, primary site of cancer, grade, stage, tumor size, laterality and histology of brain malignancy, prior cancer site, surgery, radiation therapy, and chemotherapy. Age was categorized into three groups 0–39, 40–60, and >60 years. Marital status was classified as married, single, divorced/separated, widowed, and unknown. Race was classified as White, Black, Others, and Unknown. Latency was defined as the time interval between index cancer and brain cancer diagnoses. OS, the time from brain cancer diagnosis to death due to any cause, and brain cancer‐specific survival (BCSS), the time from brain cancer diagnosis to death due to brain cancer, were calculated using vital status and cause‐specific death classification, respectively (Figure 2).

### 2.3 Statistical analysis

The patients were separated into those without prior cancer history and those with previous cancer history based on SEER sequence numbers. Descriptive statistics were applied to summarize demographic, pathological, and clinical data; the categorical variables were reported as number of cases and percentages. Baseline characteristics were compared between patients with and without prior cancer history using the Pearson Chi‐square test. Confounding due to variation in baseline characteristics was minimized using the Propensity Score Matching (PSM) method. Propensity scores were calculated based on Sex, Marital status, Year of Diagnosis, Race, Primary CNS Site, Histological Type, Tumor Grade, Laterality, Stage, Grade, Surgery, Age, Radiation, Chemotherapy and Tumor Size and a one‐to‐one PSM was employed to match patients with prior cancer history to those without prior cancer history by utilizing the nearest neighbor method with a caliper distance of 0.2. A histogram of standardized differences before and after PSM was plotted to visually exhibit the properties of the matching procedure. The Log‐rank test and Gray's test were used to compare OS and BCSS in patients with and without a prior cancer history, respectively, and Kaplan–Meier curves were constructed. The multivariable Cox regression and Fine‐and‐Gray competing risk regression analyses were used to determine the impact of prior cancer history on OS and BCSS, respectively. All statistical analyses were undertaken in R software version 4.2.2.

## 3 RESULTS

### 3.1 Patient demographics and baseline characteristics

A total of 42 726 eligible patients with brain cancer were included, of whom 1189 (2.8%) had a prior cancer history. The median time interval (IQR) between the index brain cancer and the prior cancer was 40 (2–118) months. We observed a higher frequency of prior cancer among specific demographic groups. Males accounted for 38.5% of patients with prior cancer, while elderly patients aged over 60 represented 47.2%. Among patients with a history of cancer, 55.9% were Caucasians, and 38.6% were married. The most common histological subtype in patients with prior cancer history was Glioblastoma (46.7%). Table 1 demonstrates the baseline demographic characteristics of patients. (Table 1) Following the adjustment of propensity scores, all variables pertaining to patients with and without a history of cancer were adequately balanced (*p* value >.05 for all).

**TABLE 1:** Baseline characteristics of brain cancer patients with and without prior cancer history in unmatched and matched datasets.

Characteristics	Original data set			Matched data set		
	NO prior cancer *N* = 41 537 (%)	With prior cancer *N* = 1189 (%)	*p* value	NO prior cancer *N* = 1188 (%)	With prior cancer *N* = 1188 (%)	*p* value
Age (years)			<.001*			.585
0–39	11 968 (28.81%)	50 (4.2%)		40 (3.37%)	50 (4.2%)	
40–60	12 703 (30.582%)	246 (13.023%)		231 (12.235%)	246 (13.030%)	
>60	16 866 (40.605%)	893 (47.274%)		917 (48.570%)	892 (47.246%)	
Gender			.002*			.768
Male	23 501 (56.578%)	727 (38.486%)		733 (38.824%)	726 (38.453%)	
Female	18 036 (43.422%)	462 (24.457%)		455 (24.100%)	462 (24.470%)	
Year of Dx			<.001*			.582
2010–1014	20 770 (50.004%)	334 (17.681%)		322 (17.055%)	334 (17.691%)	
2015–2019	20 767 (49.996%)	855 (45.262%)		866 (45.869%)	854 (45.233%)	
Race	(0.000%)	(0.000%)	.001*	(0.000%)	(0.000%)	.865
White	35 520 (85.514%)	1056 (55.903%)		1065 (56.409%)	1055 (55.879%)	
Black	2832 (6.818%)	78 (4.129%)		74 (3.919%)	78 (4.131%)	
Others	2874 (6.919%)	53 (2.806%)		48 (2.542%)	53 (2.807%)	
Unknown	311 (0.749%)	2 (0.106%)		1 (0.053%)	2 (0.106%)	
Marital status			<.001*			.761
Married	21 077 (50.743%)	729 (38.592%)		720 (38.136%)	729 (38.612%)	
Single	13 016 (31.336%)	155 (8.205%)		172 (9.110%)	155 (8.210%)	
Divorced/Separated	3106 (7.478%)	95 (5.029%)		92 (4.873%)	95 (5.032%)	
Widowed	2663 (6.411%)	68 (3.600%)		75 (3.972%)	68 (3.602%)	
Unknown	1675 (4.033%)	142 (7.517%)		129 (6.833%)	141 (7.468%)	
Histological type			<.001*			.68
Glioblastoma	22 585 (54.373%)	882 (46.691%)		923 (48.888%)	882 (46.716%)	
Astrocytoma	5532 (13.318%)	105 (5.558%)		87 (4.608%)	105 (5.561%)	
Glioma^a^	3695 (8.896%)	65 (3.441%)		55 (2.913%)	64 (3.390%)	
Oligodendroglioma	2539 (6.113%)	46 (2.435%)		42 (2.225%)	46 (2.436%)	
Other	7186 (17.300%)	91 (4.817%)		81 (4.290%)	91 (4.820%)	
Grade			.02*			.92
Grade I	766 (1.844%)	14 (0.741%)		15 (0.794%)	13 (0.689%)	
Grade II	1569 (3.777%)	26 (1.376%)		27 (1.430%)	26 (1.377%)	
Grade III	976 (2.350%)	29 (1.535%)		25 (1.324%)	29 (1.536%)	
Grade IV	11 338 (27.296%)	343 (18.158%)		328 (17.373%)	343 (18.167%)	
Unknown	26 888 (64.733%)	777 (41.133%)		793 (42.002%)	777 (41.155%)	
Stage			.005*			.99
Stage I	32 504 (78.253%)	957 (50.662%)		959 (50.794%)	956 (50.636%)	
Stage II	5938 (14.296%)	157 (8.311%)		153 (8.104%)	157 (8.316%)	
Stage III	847 (2.039%)	8 (0.424%)		9 (0.477%)	8 (0.424%)	
Unknown	2248 (5.412%)	67 (3.547%)		67 (3.549%)	67 (3.549%)	
Surgery			.072*	(0.000%)		.78
Yes	31 449 (75.713%)	874 (46.268%)		889 (47.087%)	874 (46.292%)	
No	9874 (23.772%)	312 (16.517%)		296 (15.678%)	311 (16.472%)	
Unknown	214 (0.515%)	3 (0.159%)		3 (0.159%)	3 (0.159%)	
Radiation			.016*			.93
No	13 963 (33.616%)	360 (19.058%)		357 (18.909%)	359 (19.015%)	
Yes	27 574 (66.384%)	829 (43.886%)		831 (44.015%)	829 (43.909%)	
Chemotherapy			.891			.9
No	16 676 (40.147%)	475 (25.146%)		477 (25.265%)	474 (25.106%)	
Yes	24 861 (59.853%)	714 (37.798%)		711 (37.659%)	714 (37.818%)	
Size			<.001*			.13
<4 cm	13 638 (32.833%)	702 (37.163%)		683 (36.176%)	701 (37.129%)	
>4 cm	19 625 (47.247%)	341 (18.052%)		364 (19.280%)	341 (18.061%)	
Unknown	8274 (19.920%)	146 (7.729%)		142 (7.521%)	146 (7.733%)	
Laterality			<.001*			.53
Left	16 025 (38.580%)	488 (25.834%)		522 (27.648%)	487 (25.794%)	
Right	16 443 (39.586%)	540 (28.587%)		508 (26.907%)	540 (28.602%)	
Not a paired site	7841 (18.877%)	125 (6.617%)		123 (6.515%)	125 (6.621%)	
Bilateral single primary	562 (1.353%)	18 (0.953%)		18 (0.953%)	18 (0.953%)	
Only one side, unspecified	53 (0.128%)	0 (0.000%)		2 (0.106%)	0 (0.000%)	
Paired site, unspecified	485 (1.168%)	17 (0.900%)		12 (0.636%)	17 (0.900%)	
Paired site, midline tumor	128 (0.308%)	1 (0.053%)		2 (0.106%)	1 (0.053%)	
Primary site			<.001*			.96
Cerebrum	1923 (4.630%)	43 (2.276%)		45 (2.383%)	43 (2.278%)	
Frontal lobe	11 398 (27.441%)	353 (18.687%)		347 (18.379%)	353 (18.697%)	
Temporal lobe	8458 (20.363%)	286 (15.140%)		290 (15.360%)	285 (15.095%)	
Parietal lobe	4881 (11.751%)	171 (9.052%)		172 (9.110%)	171 (9.057%)	
Occipital lobe	1207 (2.906%)	54 (2.859%)		60 (3.178%)	54 (2.860%)	
Ventricles	766 (1.844%)	8 (0.424%)		11 (0.583%)	8 (0.424%)	
Cerebellum	2476 (5.961%)	24 (1.271%)		22 (1.165%)	24 (1.271%)	
Brainstem	2170 (5.224%)	16 (0.847%)		22 (1.165%)	16 (0.847%)	
Overlapping	4478 (10.781%)	130 (6.882%)		130 (6.886%)	1 (6.886%)	
Not otherwise specified	3780 (9.100%)	104 (5.506%)		89 (4.714%)	104 (5.508%)	

^a^ Glioma: to refer the grade I, grade II, and grade III glial cell tumors.

* Indicates *p* value less than .05.

### 3.2 Impact of previous cancer history on the survival of patients with brain cancer

Patients with and without PCH had a median OS of 8 and 19 months, respectively. Following PSM, the OS for patients with PCH remained 8 months, whereas patients without PCH had a reduced OS of 11 months.

**FIGURE 3:**
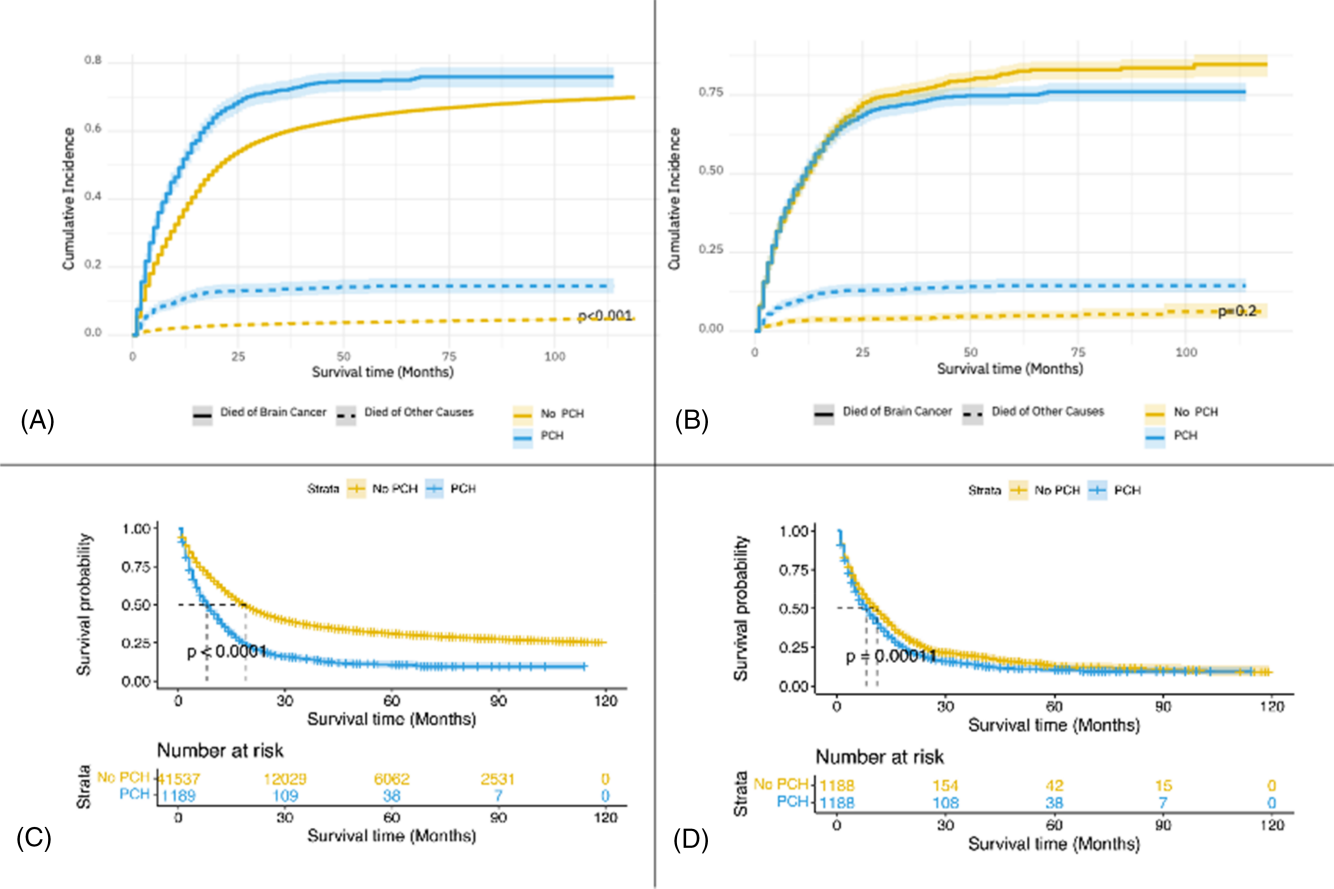
Cumulative incidence and Kaplan–Meier survival curves illustrating the effect of prior cancer history on the overall survival (OS) of brain cancer patients before and after PSM (A: Cumulative incidence of death in patients with and without PCH before PSM. B: Cumulative incidence of death in patients with and without PCH after PSM. C: KM curve of patients with and without PCH before PSM. D: KM curve of patients with and without PCH after PSM).

Cumulative incidence and Kaplan–Meier curves were generated to illustrate the survival outcomes of brain cancer patients with and without PCH (Figure 3). The cumulative incidence of death was notably higher in patients with PCH compared to those without (Figure 3A). However, due to PSM, the cumulative incidences of the two groups became similar (*p* value = 0.2) (Figure 3B). PCH had a detrimental impact on the survival of patients (*p* value <.001) (Figure 3C). Nevertheless, after PSM, the survival curves converged, indicating comparable outcomes (Figure 3D).

The result of multivariate Cox regression analysis for OS and BCSS is listed in the Table 2. Age, Sex, Race, Marital status, Histological type, Tumor size, Grade, Stage, Surgery, Radiation, Chemotherapy, Latency, Year of diagnosis, and Organ system of previous cancer were entered into the model for OS and subsequently CSS. The overall models were statistically significant (*p* value <.001).

**TABLE 2:** Multivariate cox‐regression analysis of overall survival (OS) and brain cancer‐specific survival (BCSS) in brain cancer patients.

Characteristic	Overall survival (OS)		Brain cancer‐specific survival (BCSS)	
	AHR (95% CI)	*p* value	AHR (95% CI)	*p* value
All patients after PSM				
Primary brain cancer	1.0	‐	1.0	‐
Second primary brain cancer	1.24 (1.12–1.36)	<.001*	0.92 (0.83–1.02)	.11
Subgroup with prior cancer				
Age (years)				
0–39	1.0	‐	1.0	‐
40–60	2.12 (1.27–3.55)	<.001*	1.73 (1.01–2.97)	.045*
>60	4.00 (2.41–6.66)	<.001*	2.65 (1.56–4.48)	<.001*
Gender				
Female	1.0	‐	1.0	‐
Male	1.00 (0.85–1.19)	.97	1.05 (0.87–1.26)	.62
Race				
White	1.0	‐	1.0	‐
Black	1.11 (0.85–1.45)	.45	0.94 (0.68–1.30)	.72
Others/unknown	0.89 (0.64–1.25)	.51	1.13 (0.81–1.57)	.46
Marital status				
Married	1.0	‐	1.0	‐
Single	1.19 (0.96–1.49)	.12	1.09 (0.85–1.40)	.48
Divorced/separated	1.17 (0.90–1.51)	.23	1.11 (0.82–1.48)	.51
Widowed	1.09 (0.81–1.47)	.57	1.05 (0.72–1.54)	.81
Unknown	1.56 (1.26–1.92)	<.001*	1.42 (1.14–1.77)	.002*
Histological type				
Glioblastoma	1.0	‐	1.0	‐
Astrocytoma	0.53 (0.40–0.70)	<.001*	0.65 (0.48–0.87)	.004 *
Glioma^a^	0.57 (0.40–0.81)	<.001*	0.71 (0.46–1.08)	.11
Oligodendroglioma	0.24 (0.15–0.39)	<.001*	0.18 (0.09–0.33)	<.001*
Other	0.37 (0.26–0.52)	<.001*	0.27 (0.16–0.46)	<.001*
Grade				
Grade I	1.0	‐	1.0	‐
Grade II	1.14 (0.43–3.02)	.79	0.85 (0.27–2.68)	.78
Grade III	2.46 (0.99–6.09)	.05	1.66 (0.58–4.77)	.35
Grade IV	2.36 (1.06–5.26)	.04*	1.40 (0.52–3.83)	.52
Unknown	1.19 (0.99–4.82)	.05	1.18 (0.44–3.21)	.74
Stage				
Stage I	1.0	‐	1.0	‐
Stage II	1.39 (1.14–1.71)	<.001*	1.44 (1.17–1.77)	<.001*
Stage III	1.57 (0.69–3.61)	.28	1.96 (1.22–3.14)	.005*
Unknown	1.05 (0.76–1.45)	.76	0.85 (0.58–1.26)	.42
Surgery				
No/unknown	1.0	‐	1.0	‐
Yes	0.69 (0.58–0.81)	<.001*	0.99 (0.81–1.22)	.93
Radiation				
No	1.0	‐	1.0	‐
Yes	0.70 (0.56–0.86)	<.001*	0.73 (0.56–0.95)	.017*
Chemotherapy				
No	1.0	‐	1.0	‐
Yes	0.55 (0.45–0.67)	<.001*	0.82 (0.65–1.02)	.076
Size				
<4 cm	1.0	‐	1.0	‐
>4 cm	1.12 (0.94–1.35)	.21	1.09 (0.90–1.33)	.39
Unknown	1.01 (0.80–1.28)	.91	1.07 (0.83–1.38)	.58
Organ system				
Genitourinary	1.0	‐	1.0	‐
Breast	1.02 (0.79–1.32)	.88	0.88 (0.66–1.17)	.37
Hematological and lymphatic	1.31 (1.05–1.63)	.01*	1.19 (0.96–1.49)	.12
Gastrointestinal	1.17 (1.93–1.46)	.18	0.85 (0.65–1.11)	.24
Skin	0.75 (0.58–0.97)	.03*	0.93 (0.71–1.22)	.59
Respiratory	1.24 (0.92–1.65)	.15	0.52 (0.35–0.78)	.002*
Endocrine	0.97 (0.67–1.41)	.87	1.19 (0.83–1.72)	.35
Unknown	1.06 (0.76–1.50)	.72	0.83 (0.57–1.19)	.31
Latency				
<12 months	1.0	‐	1.0	‐
12–36 months	1.04 (0.87–1.26)	.65	1.27 (1.03–1.57)	.023*
37–60 months	1.00 (0.80–1.24)	.99	1.24 (0.98–1.57)	.073
>60 months	1.04 (0.81–1.32)	.76	1.23 (0.94–1.62)	.13
Year of Dx				
2010–1014	1.0	‐	1.0	‐
2015–2019	0.90 (0.76–1.09)	.30	0.87 (0.72–1.05)	.15
Primary site				
Cerebrum	1.0	‐	1.0	‐
Frontal lobe	0.89 (0.62–1.28)	.54	0.82 (0.58–1.17)	.28
Temporal lobe	0.77 (0.53–1.10)	.16	0.77 (0.54–1.09)	.14
Parietal lobe	0.86 (0.59–1.26)	.44	0.93 (0.64–1.34)	.70
Occipital lobe	0.72 (0.46–1.16)	.18	0.65 (0.41–1.03)	.06
Cerebellum	0.76 (0.40–1.47)	.42	0.60 (0.30–1.22)	.16
Brainstem	0.50 (0.24–1.05)	.07	0.76 (0.35–1.66)	.50
Overlapping	0.97 (0.66–1.44)	.90	0.82 (0.56–1.22)	.33
Not otherwise specified	1.04 (0.69–1.58)	.85	0.96 (0.62–1.50)	.86

^a^ Glioma: to refer the grade I, grade II, and grade III glial cell tumors.

* Indicates *p* value less than .05.

Cox Regression analysis revealed PCH to be a significant independent risk factor for the OS of patients. (aHR 1.26; 95% CI 1.15–1.39). However, second primary to PSM, the BCSS of patients with and without prior cancer history was comparable (subdistribution Hazard Ratio SHR 0.97; 95% CI 0.88–1.07, *p* value .54).

In the subset of patients with PCH, older age (>60 years) had a significant prognostic impact on the OS (aHR 4.00; 95% CI 2.41–6.66, *p* value <.01) and BCSS (SHR 2.65; 95% CI 1.56–4.48, *p* value <.01) of patients.

Glioblastoma exhibited the most substantial and statistically significant impact on OS and CSS as compared to other histological types.

Early‐stage 2 prior cancers were significant risk factors for OS (AHR 1.39; 95% CI 1.14–1.71, *p* value <.01) and BCSS (SHR 1.44; 95% CI 1.17–1.77, *p* value <.01) compared to advanced stages.

Patients who did not undergo surgery for tumor removal exhibited a notably lesser OS compared to those who underwent surgery (AHR 0.69; 95% CI 0.58–0.81, *p* value <.01). However, it is important to note that tumor removal surgery did not demonstrate a significant influence on the BCSS (SHR 0.99; 95% CI 0.81–1.22, *p* value = .93). (Table 2).

### 3.3 Prior cancer types

**FIGURE 4:**
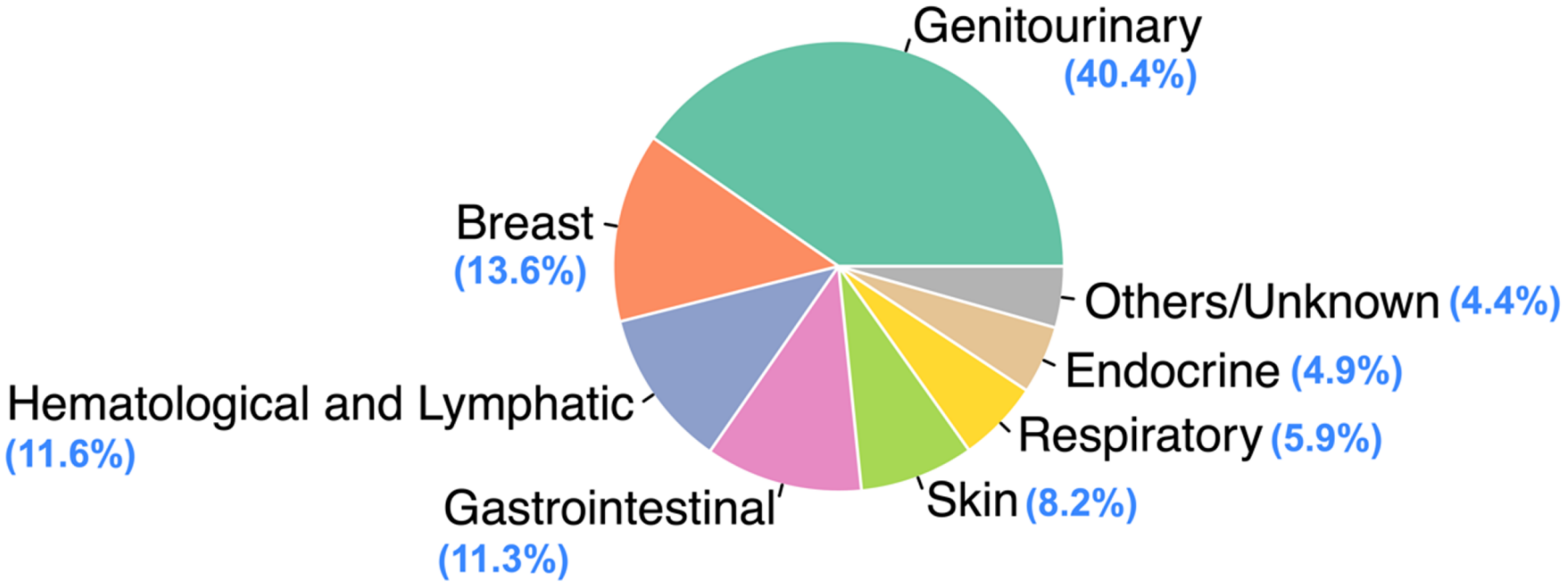
Pie chart demonstrating distribution of prior cancer types in various organ systems.

Prior cancers were most reported from Genitourinary (40.4%), Breast (13.6%), Hematologic and Lymphatic (11.4%), and Gastrointestinal malignancies (11.3%) (Figure 4).

The OS varied significantly among brain cancer patients who had different types of prior cancer (*p* value .001). Patients with prior gastrointestinal malignancies had the shortest median OS (6 months) followed by Hematological and lymphatic cancers (7 months).

**FIGURE 5:**
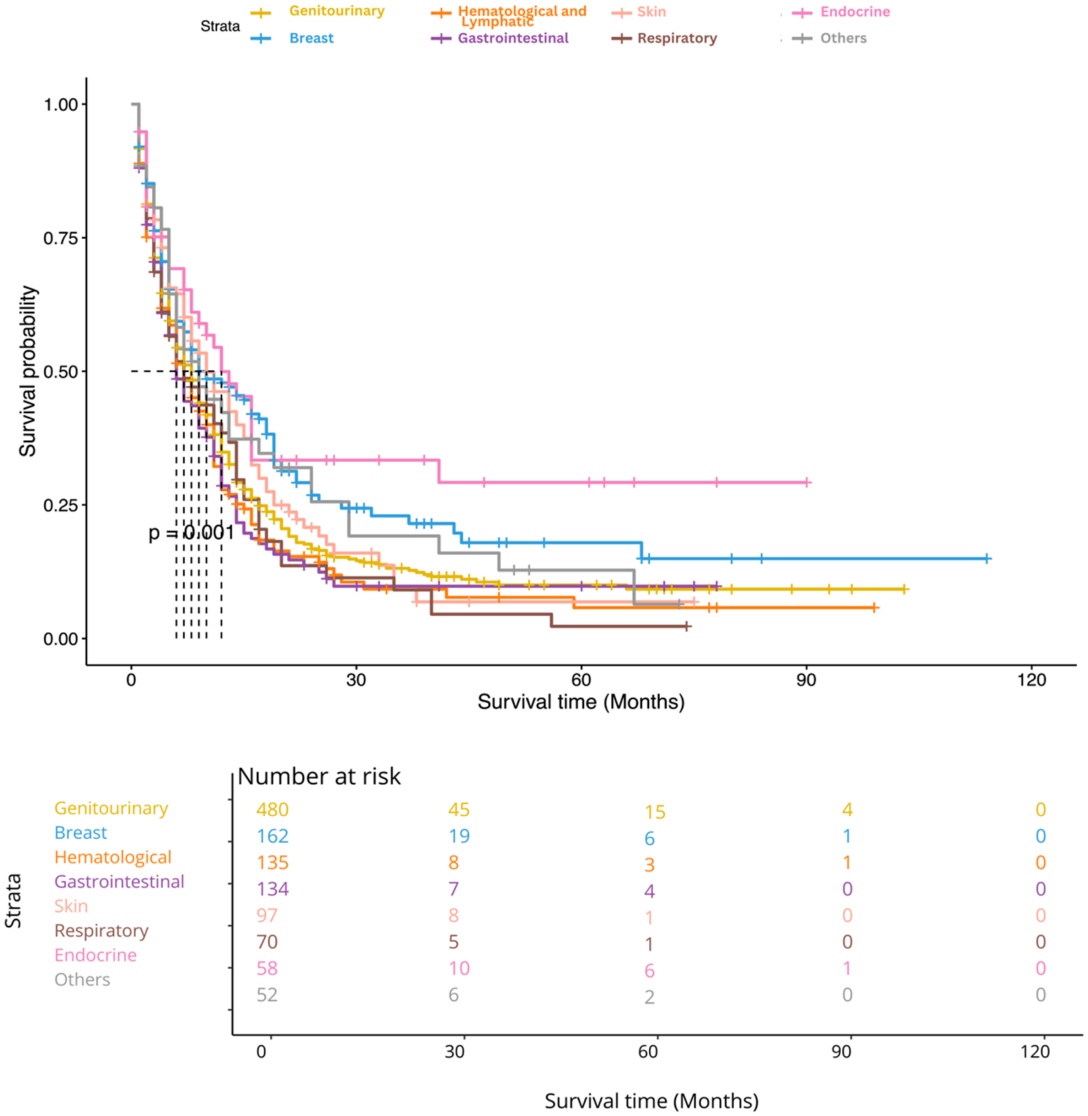
Site specific survival of patients with previous cancer history.

Figure 5 illustrates the overall survival (OS) of patients with brain cancer who had different types of prior malignancies, as determined by an adjusted Kaplan–Meier curves of the matched dataset.

## 4 DISCUSSION

Limited data exists on the risk factors associated with brain malignancies due to their rare occurrence, constituting 1% of newly diagnosed cancers. To our knowledge, there exists a paucity of literature in patients with prior cancer diagnoses.^9^ In this study, we identified 1189 patients with second primary brain cancer and prior cancer history using the Surveillance, Epidemiology, and End Results (SEER) registry, which accounts for approximately 48% of the US population.^10^ The median time interval between diagnoses of index brain cancer and the prior cancer was 40 months. Brain cancer after previous cancer history is associated with male patients, patients >60 years of age, Caucasian patients, and patients who were married. The most common prior cancer diagnoses were genitourinary (40.4%), breast (13.6%), hematologic and lymphatic (11.4%), and gastrointestinal (11.3%). Gastrointestinal malignancy was associated with the shortest OS time of 6 months. The median OS for patients with prior cancer history was 8 months. Prior cancer history was found to be an independent risk factor for OS in this patient population, with older age had an impact on OS. Glioblastoma was the most prevalent brain cancer in the study population.

Although the exact association is not understood, there are several working hypotheses which posit prior cancer history as a risk factor. A common hypothesis is the use of chemotherapy and radiation therapy in cancer patients. There has been ample data reporting that cranial radiation therapy is a key risk factor in developing subsequent CNS tumors, especially high‐grade meningiomas and gliomas, in survivors of cancer.^11^, ^12^, ^13^, ^14^, ^15^ Similarly, in a retrospective study of patients who developed second primary glioma, Maluf et al.^16^ found that most of the patients had received either prior chemotherapy and/or radiation preceding glioma diagnosis. Administration of intravenous and intrathecal methotrexate has also been associated with development of meningiomas,^17^, ^18^ as well as alkylating agents such as bleomycin and chloraminophen.^19^ Although the association of radiation and brain cancer is stronger in the pediatric population, the incidence of second primary brain neoplasms post‐radiation in adults ranges from 2.7% to 8.5% with a latency period of 5–34 years.^20^

Another common hypothesis is one of shared genetic pathways: several studies have investigated genetic predispositions which increased susceptibility to both primary cancers and subsequent brain malignancies. In a population‐based study, Li et al.^21^ reported that 17.8% of patients with glioma whose tumor had a *TP53* mutation also had a history of prior cancer. In a study evaluating subsequent neoplasms in children, Turcotte et al.^22^ found that primary cancer may beget susceptibility syndromes, many of which exhibit autosomal dominant inheritance pattern. This can give rise to CNS cancers, such as gliomas.

Most recently, Liu et al.^23^ analyzed patients records from 1975 to 2016 assessing the risk of second primary neoplasms of the CNS. Like our findings, glioblastomas represented a plurality of CNS cancers observed and gave rise to worse relative outcomes. In addition, somewhat similar to our findings, Liu et al.^24^ found the most common primary sites to be genitourinary (29.2%), breast (17.6%), gastrointestinal (17.4%), and lung (10.8%). Cranial radiation therapy was identified as a strong risk factor. In another population‐based study, Wang et al. investigated whether the prevalence of primary brain tumors was associated with systemic malignancies. The most common primary sites were prostate cancer (28.2%), breast cancer (14.9%), or melanoma of the skin (11.3%), and breast cancer had a lower risk of brain cancer development, which contrasts with our findings of genitourinary (40.4%), breast (13.6%), and hematologic and lymphatic (11.4%) as the most common primary sites. The median survival time was 6 months compared to our finding of 8 months. While our data suggests chemotherapy and radiation therapy had prognostic value to OS, Wang et al. found no difference in incidence ratios for patients who underwent radiation or chemotherapy.

Currently, there is a shortage of data on OS in this population. In patients with a history of gastrointestinal cancer and subsequent brain metastasis, OS has ranged from 3.2 to 8 months, compared to the reported findings of 6 months.^25^, ^26^, ^27^ Less data is presented for prior hematologic/lymphatic cancers. One retrospective cohort study of patients with non‐Hodgkin lymphoma and second primary CNS involvement found a median survival time of 11 months, which differs from the 7 months found here.^28^ These findings contextualize the importance of inclusion of patients with second primary brain cancer in ongoing clinical trials. In an analysis of 464 clinical trials, more than one‐third excluded this population completely, with another one‐third instituting conditional exclusion criteria.^29^ The exclusion from phase III trials has been largely justified by the poor life expectancy, prognostic factor, heightened vulnerability, and limited therapeutic tolerability.^30^ Ergo, we urge greater inclusion and diversification of patients with second primary brain cancer to increase generalizability of results and treatment strategies, while imploring caution in patients with prior history of gastrointestinal or hematologic/lymphatic cancer.

Our study has a few limitations. First, the study design is observational and retrospective in nature, which may limit the ability to distinguish correlation from causation between variables. Second, the SEER database, albeit commonly used, presents geographic, variable, and time limitations which may give rise to selection bias. Third, the data extracted in this study does not report the extent or staging of the malignancies being studied and may also lack clinical detail on the treatment modalities, such as surgery, radiation therapy, and chemotherapy. However, we aimed to reduce the influence of these limitations and biases by propensity matching cohorts and reporting on multiple variables to provide different dimensions to our findings. It is important to note that the breakdown of primary cancers in this study is presented based on histological classifications rather than specific tumor sites. While we recognize the potential benefits of analyzing cancers by tumor site, our study design focused on histology as a primary categorization.

### 4.1 Limitations related to study populations

1. Inherent challenges in comparing patients with prior cancer history to naïve cancer patients:

- Introduction:
  - Despite the rigor and relevance of our analysis, it is essential to acknowledge inherent limitations associated with comparing patients with a history of prior cancer to those without any such history (naïve patients).
  - The primary objective of our study is to explore the impact of prior cancer history on survival in brain cancer patients, yet the interpretative landscape is complex due to fundamental differences in epidemiologic characteristics between these two groups.
- Epidemiologic distinctions:
  - Clinical trials commonly exclude patients with prior cancer history, not due to a lack of evidence, but rather as a response to basic epidemiologic distinctions between these populations.
  - These distinctions can be confounding factors that may influence the interpretation of our findings, and it is crucial to acknowledge the potential limitations associated with this inherent heterogeneity.

2 Genetic differences and impact of serious genetic alterations:

- Genetic nature of double primary cancers:

It is essential to recognize that double primary cancers are often genetic diseases with complex pathophysiologies rooted in substantial genetic alterations.

b Focus on survival outcomes:

Our study primarily focuses on survival outcomes in brain cancer patients with prior cancer history, rather than providing an exhaustive exploration of the underlying genetic mechanisms.

While we acknowledge the significance of genetic differences, our study is centered on contributing valuable insights into survival dynamics within this specific patient population.

c Limitation of detailed genetic exploration:

The study, by design, does not delve into the detailed genetic mechanisms of cancer genesis in double primary cancers.

Recognizing the importance of this aspect, we emphasize that our primary goal is to provide valuable contributions to the understanding of survival outcomes.

3 Exploring comprehensive insights

The diverse range of cancer types allows for a holistic examination, ensuring that our analysis captures a broad spectrum of potential influences on survival outcomes in brain cancer patients with prior cancer history.

We recognize the complexity introduced by this diversity and acknowledge it as a potential limitation, emphasizing the trade‐off between inclusivity and specificity in our analysis.

## 5 CONCLUSION

Our findings determines that PCH does not have a significant impact on the survival of brain cancer patients, except for gastrointestinal or hematologic and lymphatic PCH or when brain cancer was glioblastoma. We suggest that all other patients could be included in clinical trials regardless of their cancer history. However, there is still a need for further prospective studies.

## AUTHOR CONTRIBUTIONS

**Mohammad Ebad Ur Rehman:** Conceptualization (equal); formal analysis (equal); methodology (equal); supervision (equal); writing – original draft (equal); writing – review and editing (equal). **Fatima Faraz:** Methodology (equal); writing – original draft (equal); writing – review and editing (equal). **Huzaifa Ahmad Cheema:** Writing – original draft (equal); writing – review and editing (equal). **Omer S. Ashruf:** Writing – original draft (equal); writing – review and editing (equal). **Hamna Raheel:** Writing – original draft (equal); writing – review and editing (equal). **Syeda Zainab Ali Naqvi:** Writing – original draft (equal); writing – review and editing (equal). **Nimrah Jabeen:** Writing – original draft (equal); writing – review and editing (equal). **Areesha Abid:** Writing – original draft (equal); writing – review and editing (equal). **Haris Mumtaz Malik:** Writing – original draft (equal); writing – review and editing (equal). **Ahmad Iftikhar:** Writing – original draft (equal); writing – review and editing (equal). **Ahmed Ibrahim:** Writing – original draft (equal); writing – review and editing (equal). **Sarya Swed:** Supervision (equal); writing – review and editing (equal).

## CONFLICT OF INTEREST STATEMENT

The authors have stated explicitly that there are no conflicts of interest in connection with this article.

### ETHICS STATEMENT

Ethical approval was not required for the study involving humans in accordance with the local legislation and institutional requirements. Written informed consent to participate in this study was not required from the participants in accordance with the national legislation and the institutional requirements.

## Data Availability

Data is available upon responsible request from the corresponding author.
